# Adaptive‐driven CT simulation‐free multi‐fraction SBRT radiotherapy: Initial clinical experience

**DOI:** 10.1002/acm2.70147

**Published:** 2025-07-25

**Authors:** V. N. Malkov, B. J. Kemp, A. Ferrero, L. Buchholtz, S. S. Park, J. A. Kavanaugh

**Affiliations:** ^1^ Department of Radiation Oncology Mayo Clinic Rochester Minnesota USA; ^2^ Department of Radiology Mayo Clinic Rochester Minnesota USA

**Keywords:** adaptive, computed tomography (CT), ethos, hypersight, simulation free

## Abstract

**Introduction:**

Using diagnostic CT for radiotherapy (RT) planning can bypass traditional CT simulation but introduces challenges in patient positioning and Hounsfield unit (HU) fidelity, affecting dose accuracy. Here a Varian Ethos adaptive‐driven CT direct‐to‐treatment (DtT) multi‐fraction stereotactic body radiation therapy (SBRT) workflow is presented.

**Methods:**

This study employed institutional diagnostic PET‐CT images to generate an initial reference Ethos treatment plan. HU and dosimetric accuracy were validated for PET‐CT, Ethos CBCT images (with and without Hypersight (HS), and the gold‐standard helical CT simulators). Following the SBRT reference plan creation on the low dose diagnostic PET‐CT, the first fraction was delivered with a newly generated adaptive plan calculated on the HS CBCT (Ethos) images. For multi‐fraction treatments, the first day CBCT images and adaptive plan become the reference for subsequent IGRT treatments. This study includes workflow validation and initial three patient experience.

**Results:**

The DtT adaptive SBRT workflow was successfully implemented, with initial end‐to‐end testing demonstrating feasibility. In‐house solutions were introduced to facilitate the adaptive to IGRT plan conversion. The Ethos system, especially with HS, maintained HU fidelity and dose calculation accuracy comparable to helical CTs. On‐table adaptive sessions were within 37–51 min, aligning with single‐fraction palliative studies. Subsequent non‐adaptive IGRT fractions were efficiently completed within 7–27 min.

**Conclusions:**

This study demonstrates the feasibility of DtT adaptive‐driven multifraction SBRT using diagnostic PET‐CT. This approach reduces the need for on‐site patient presence prior to treatment, expands planning lead times, and improves overall efficiency in radiotherapy for well selected patients.

## 1 INTRODUCTION

The standard radiotherapy planning and delivery process involves CT simulation in the treatment position. Avoiding the CT simulation in well‐selected patients can reduce patient costs and improve quality of life metrics.^1^, ^2^, ^3^, ^4^, ^5^ Patient selection criteria can depend on the treatment technique but include patient tolerance for on‐couch time, setup challenges, potential time saving, and quality of available diagnostic images. Challenges with direct‐to‐treatment (DtT) stereotactic body radiation therapy (SBRT) planning include potential discrepancies in Hounsfield Unit (HU) between the low dose PET‐CTs and helical simulation CTs and the patient setup variation which may introduce dosimetric uncertainties. However, DtT workflow for palliative treatments can reduce consult to treatment times.^6^, ^7^, ^8^ Therefore, careful patient selection and interdisciplinary review between physicians, medical physicists, dosimetrists, and therapists are crucial to successfully implement a DtT SBRT program.

SBRT is becoming widely utilized in radiation treatments, especially in oligometastatic and retreatment settings.^9^, ^10^, ^11^, ^12^ Key aspects of SBRT treatments are precise target delineation, high doses per fraction with steep dose gradients away from the target, accurate treatment image matching, and patient motion management or immobilization. Conventional CBCT‐based DtT workflows have limited applications in the context of SBRT treatments. With the introduction of on‐line adaptive radiotherapy systems, DtT workflows that account for patient anatomy variations become feasible.^13^, ^14^, ^15^, ^16^ The Ethos (Varian, a Siemens Healthineers Company, Palo Alto, CA) system facilitates adaptive radiotherapy treatments (ART) using rapid CBCT imaging coupled with semi‐automated contouring and automated on‐unit treatment optimization.^17^, ^18^, ^19^, ^20^ The on‐board CBCT imaging grants high HU fidelity, image quality, and dose calculation accuracy.^21^, ^22^ Further, the updated imaging system, Hypersight (HS), provides a larger field‐of‐view, reduction in image acquisition time and improvement in HU reproducibility and image quality.^23^ Given the capabilities of the Ethos system, both HS and non‐HS, the FAST‐METS trial introduced a DtT adaptive process for palliative single fraction treatments^13^ and MacDonald et al. have demonstrated the feasibility of an adaptive DtT workflow using a reference Hypersight CBCT image.^24^

Herein, a HS Ethos‐based workflow for multi‐fraction DtT SBRT treatments using institutional PET‐CT diagnostic images is presented. A single Ethos adaptive fraction accounts for the patient geometry difference from the PET‐CT and establishes the conventional image guided radiotherapy (IGRT) plan for subsequent fractions. This process is unique from previous implementations in allowing for transitioning to IGRT fractions with the intent of reducing on machine time. Comparatively, FAST‐METS focused on single fraction palliative treatments and MacDonald et al. used a reference HS CBCT image to drive a same day workflow. Patients with bone met targets are selected in the current work to facilitate on‐unit contouring time and reduce considerations for motion encompassing techniques in this initial cohort. HU accuracy of institutional images and Ethos CBCTs (HS and non‐HS) is evaluated. Further dose metrics are intercompared for images used in the DtT workflow and subsequent initial clinical experience for a three‐patient cohort.

## 2 METHODS

In the DtT process, the radiation oncologist used institutional PET‐CT for delineating target and OAR contours. The initial adaptive fraction accounts for patient setup and anatomical differences, and this new adaptive plan on the first fraction CBCT is the reference for subsequent IGRT fractions. Here, the overall DtT workflow is outlined followed by, HU and dose validation, full workflow testing, and initial patient experience.

### 2.1 Workflow

**FIGURE 1:**
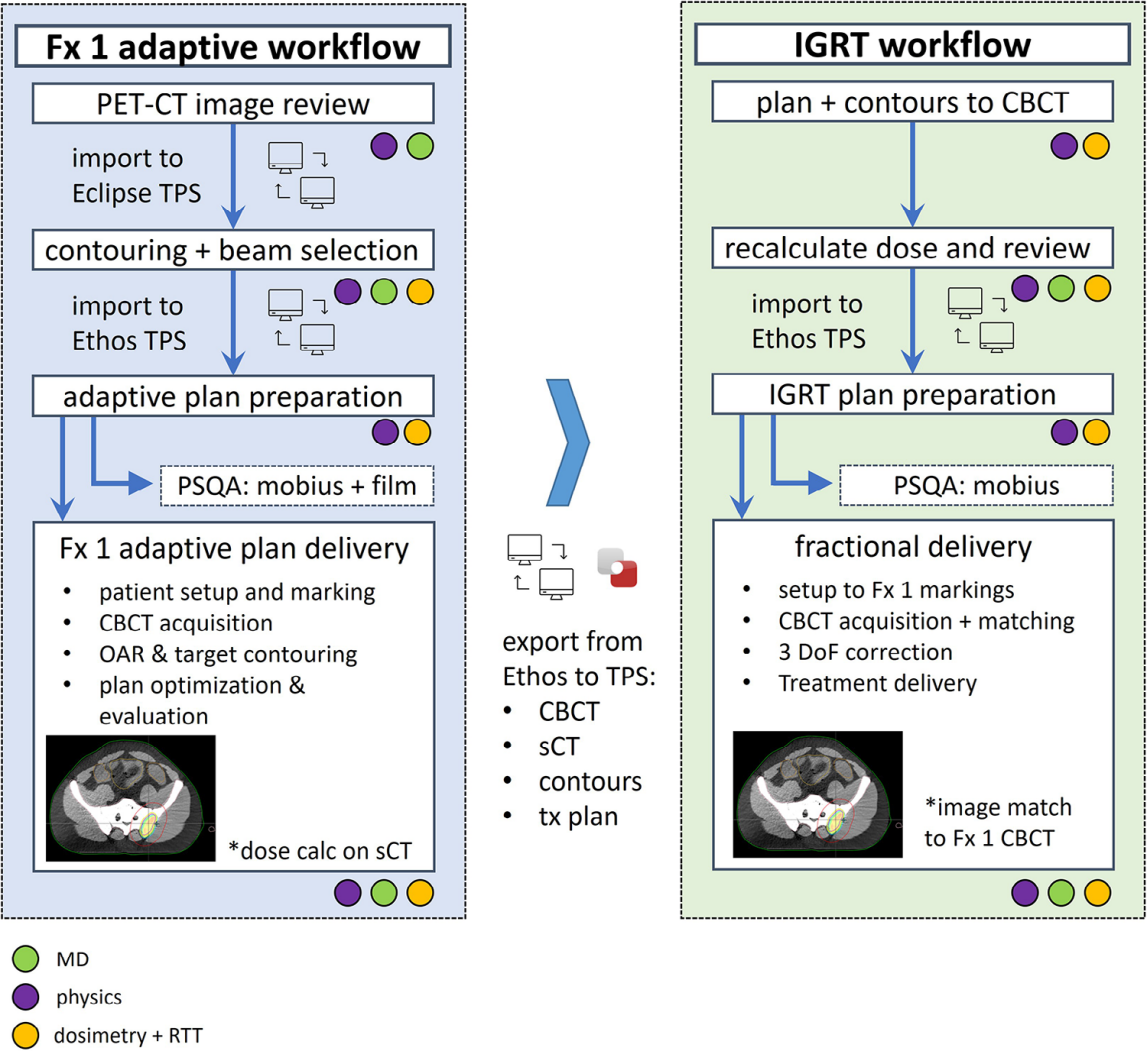
DtT workflow summary with adaptive fraction components presented (left/blue) and subsequent IGRT fractions (green/right) PSQA is patient specific quality assurance.

The institutional picture archiving and communication system (PACS), MIM (MIM Software, Beachwood OH), Eclipse TPS (Varian, Palo Alto, CA), and Ethos Treatment Management (ETM) are used through the DtT process. The workflow, including multi‐disciplinary role involvement, is visually represented in Figure 1 and described below.

#### 2.1.1 Contouring and planning

The diagnostic images in the hospital PACS were reviewed by the physician and medical physicist to evaluate target locations, anticipated patient setup, and potential limitations of the DtT process. Patient selection criteria included tolerance for prolonged on‐table time, a maximum of two independent bony targets, and minimal imaging artifacts. Diagnostic PET‐CT images were up‐sampled to 1mm slice thickness and sent to the Eclipse TPS for target and OAR contouring. A virtual CT setup point was identified based on anatomical landmarks near the treatment site and documented for on‐machine setup instead of tattoos or surface markers. The PET‐CT and contours were transferred to ETM for adaptive planning. Beam arrangements were customized to each patient to avoid the external contour clipping due to limited CBCT field‐of‐view, considering the HS maximum reconstruction diameter of 70 cm. IMRT and VMAT plans were compared to balance optimization and delivery times. All plans underwent detailed physics checks and QA via log‐file analysis and film dosimetry.

#### 2.1.2 Adaptive treatment delivery

At the first fraction, the patient is consented, immobilized, and marked for setup reference if treating multiple fractions. Immobilization devices are documented for future fractions. Subsequently, a CBCT is acquired, and OARs and targets are contoured and reviewed. Ethos on‐couch optimizer then generates a re‐computation of the reference plan (scheduled plan) and an adapted plan. For adaptive treatment dose computation, the Ethos on‐couch optimizer uses a synthetic CT (sCT) which is generated by deformable propagation of the reference CT HU values. Following scheduled and adapted plan computation, target and OAR goals are assessed in both plans, with the final to‐be treated plan selected for maximizing plan quality. The physicist will review the automatically generated external contour, positioning of high density/boney structures, and the sCT in Mobius3D. Mobius3D provides a secondary dose calculation and the ETM will automatically export the sCT, session treatment plans and doses for evaluation. In the situation of a substantial deviation of the external or sCT from the true anatomy, the patient would be direct to a conventional process. At completion of physics QA check, a verification CBCT image is obtained prior to treatment to correct for rigid patient shift.

#### 2.1.3 Post adaptive treatment export and IGRT plan generation

The selected plan of the first fraction is exported from ETM to the Eclipse TPS. MIM facilitates CBCT DICOM header retagging to allow for import into Eclipse and selection of the CBCT image, structure set, and treatment fields. The use of MIM is not strictly necessary, as manual or in‐house solutions may be used to achieve the structure and treatment plan re‐assignment. In Eclipse, the treatment fields of the imported plan are used to re‐compute dose on the CBCT image and dose metrics are reported to the transferred contours. The recomputed dose is reviewed in Eclipse to verify transfer and dose metric agreement with the first fraction selected plan. The CBCT, structures, and treatment plan are imported into the ETM. The CT setup location is set to the first fraction markers on the CBCT. An independent physics plan review and log‐file treatment delivery QA is performed on the revised plan. Subsequent fractions are delivered with the patient setup using the marks and immobilization created at the first fraction.

### 2.2 Imaging systems and HU validation

This study utilized CT, CBCT, and sCT images from institutional systems, including PET‐CT via Siemens (Siemens Healthineers, Malvern, PA, USA) Biograph Vision and GE (GE HealthCare, Milwaukee, Wisconsin, USA) Discovery MI1 (protocols at 120/140 kVp, 2.8 mm slice thickness), and four RO‐CTs using Siemens Somatom Definition AS and X.cite (120/140 kVp, 1 mm slice thickness). Ethos default and HS systems capture CBCTs (120/140kVp, 2 mm slice thickness), with Feldkamp Davis Kress (FDK), iterative CBCT (iCBCT), iCBCT Acuros, or iCBCT metal artefact reduction (MAR) reconstructions (latter two are HS exclusive). All reconstruction modes are evaluated in this study, though only the iCBCT Acuros or iCBCT MAR are used for the clinical DtT process. Independent of the DtT process, all imaging machines are regularly utilized in our practice for simulation CT (RO‐CTs), target definition (PET‐CT), and adaptive CBCT planning, and are routinely tested to comply with image quality and geometric fidelity standards.^25^, ^26^

For adaptive treatment dose computation, ETM propagates reference CT HU values via rigid and deformable registration for sCT generation. Consequently, HU accuracy validation is necessary for all imaging systems. This was evaluated using a CIRS (Mirion Medical Company, Sun Nuclear, Melbourne, FL, USA) phantom (model 062M) on PET‐CT, CBCT, and sCT with the techniques described above. CBCT evaluation focuses on the pelvis (120 kVp) and pelvis large (140 kVp) protocols though the head (100 kVp) protocol is also evaluated. The mean and standard deviation of the HU values were extracted, using an in‐house tool, from a structure (D = 1 cm, L = 1.5 cm) centered on each of the density inserts.

### 2.3 Dosimetric validation

**FIGURE 2:**
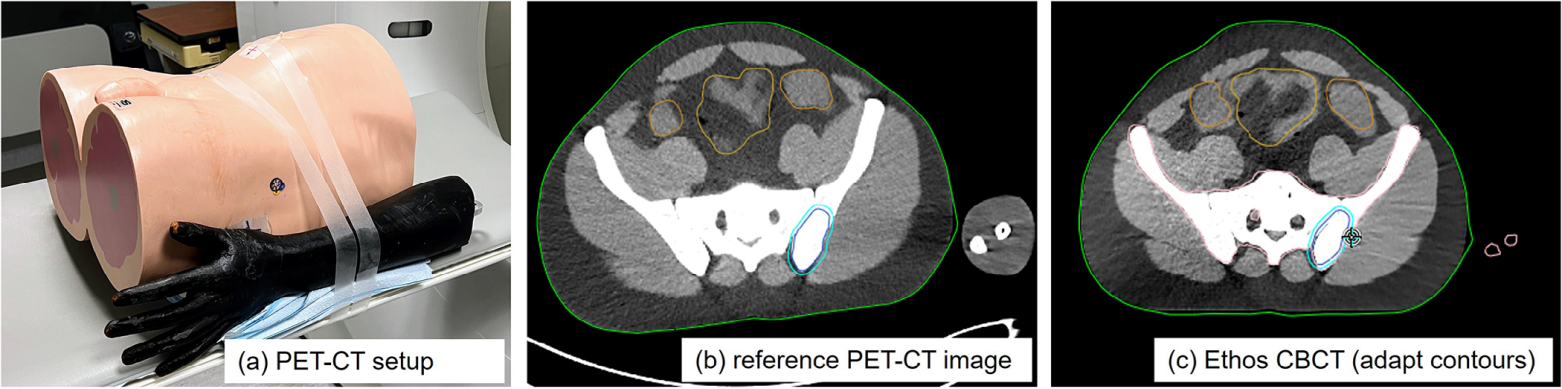
Anthropomorphic male pelvis phantom with an arm (a), the resulting PET‐CT image (b) and the Ethos CBCT image in treatment position with adapt contours (c). The arm was included in the PET‐CT scan, excluded from the external in the reference planning image, and was absent from the phantom setup for the CBCT.

An anthropomorphic male pelvis phantom (Figure 2a) was scanned using the RO‐CT, PET/CT and Ethos CBCT systems. The Ethos CBCT was collected through an adaptive workflow which provided a CBCT and an sCT of the phantom. Doismetric accuracy was evaluated by computing doses on the RO‐CT, PET/CT, Ethos sCT, and Ethos CBCT (using Pelvis CBCT with Acuros iCBCT reconstruction) images. A 2‐field partial‐arc VMAT plan was optimized with a prescription of 3000 cGy on one of the RO‐CT scans of the anthropomorphic male pelvic phantom. Images from the various systems were co‐registered and the plan was re‐computed on each image in Eclipse. Using an institutional MIM workflow, the PET‐CT images were registered to the reference RO‐CT using 6‐degrees‐of‐freedom correction and then re‐gridded. This registration eliminates setup and dose grid misalignment between the imaging systems which impact the dose comparison.

For the computed plans, the target volume, D2cc, D98%, and D95% of the PTV, the D2cc for the small and larger bowel, and the V80% were extracted (metrics are relative to 3000 cGy). Target volume is reported to confirm registration and slice thickness does not impact dose metric reporting. Individual image metrics were then compared against metric averages obtained from the RO‐CT computations.

### 2.4 Workflow testing

End‐to‐end workflow validation used the anthropomorphic pelvis phantom, scanned with the Siemens PET‐CT systems, and transferred to the Eclipse TPS. A radiation oncologist contoured OARs and a target (small bowel, larger bowel, bladder, and boney target). The CT and associated contours were transferred to the ETM, where an adaptive intent was generated that creates a PTV with 3mm margins from the defined boney target. A 9‐field IMRT reference plan was generated using defined target and OAR clinical goals for a 3000cGy in 3 fraction treatment. Goals and target metrics were used based on clinical SBRT templates and ordered using institutional clinical planning structure. Pre‐treatment QA for the reference plan was run using the Mobius3D system.

Figure 2 shows the pelvis phantom and the corresponding PET‐CT and CBCT images. The PET‐CT scan was acquired with an arm phantom attached and rolled pelvis to simulate different patient positioning and evaluate the impact on generation of the external contour during the adaptive fraction.

### 2.5 Clinical implementation experience

A cohort of three patients were treated using the DtT process with a two‐level (nested high and low dose PTVs) SBRT dose prescription (PTV = CTV + 3 mm). Table 2 provides target and prescription details. This data is being presented as a case review with all patients consenting to data usage for research purposes. Beam geometry was configured in Eclipse based on target laterality and the extent of the patient external and then imported into ETM. Pelvis or Pelvis Large techniques with Acuros iCBCT reconstruction were used for adaptive CBCT image acquisition on an HS Ethos system. The setup time (treatment consent, setup, immobilization), contouring, plan computation, plan review (including scheduled and adaptive plan computation), and time‐on‐table for the entire fraction were extracted from ETM reported timestamps and DICOM tags. Workflow components such as CBCT acquisition and reconstruction, image review, initial plan computation, ethos contour propagation, were not subdivided but contributed to the total treatment time. To verify dose agreement, the first fraction adapted plans were recomputed in Eclipse on the sCT and adaptive planning CBCT. The resulting dose distributions were then compared using 2%/2mm/10% threshold gamma as well as the D95% of the PTVs.

## 3 RESULTS

### 3.1 HU validation

**FIGURE 3:**
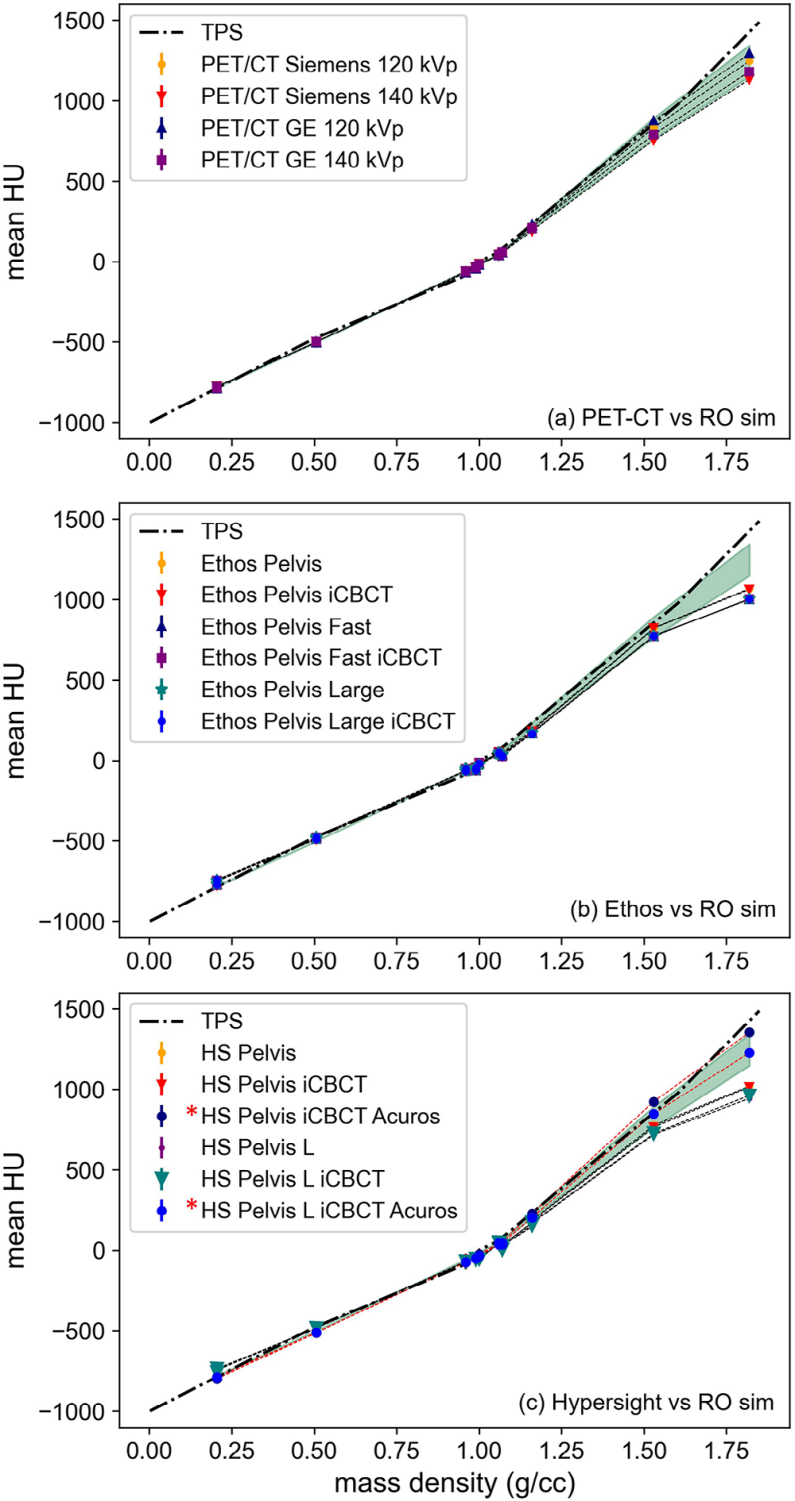
CIRS phantom Mean HU, vs electron mass density for the PET‐CT (a), non‐HS Ethos CBCT (b), and HS Ethos CBCT (c) images compared against the RO CT range presented as the background green shaded region. Black dashed‐dot line represents the TPS HU to density curved used for dose computation and is constant across (a)–(c). *Acuros reconstruction values are highlighted with a red dashed connecting line in (c).

Figure 3 shows mean HU values from the CIRS phantom across systems and representative techniques used for clinical operations. In Figure 3a, PET‐CT data taken on 2 different diagnostic systems and using 2 different techniques aligned well across the measured range of the 4 departmental RO‐CT scanners (green background range). The black dashed line represents the TPS HU‐to‐density calibration curve used for dose calculation in ETM. In Figure 3b, non‐HS Ethos CBCT imaging is shown for two different acquisition settings (Pelvis and Pelvis Large) and three different reconstruction algorithms (FDK, iCBCT, and iCBCT Acuros). The non‐HS Ethos image exhibits consistent HU fidelity at low densities, diverging beyond 1.0 g/cc. HS Ethos maintains agreement with the RO‐CTs up to 1.5 g/cc at which point only the iCBCT Acuros algorithm remains within the RO‐CT region. The iCBCT Acuros MAR reconstruction, not shown, follows the iCBCT Acuros results closely. The head scanning protocol (100 kVp), not shown, with iCBCT Acuros yielded similar HU agreement, though with increased standard deviations.

### 3.2 Dosimetric validation

Table 1 presents the dose metrics associated with the VMAT plan, initially optimized on one of the RO‐CT scans, re‐computed on all the imaging systems and compared to the average of all the RO‐CT metrics. The Ethos HS CBCT image utilized the Pelvis Large with iCBCT Acuros reconstruction. The differences between PET‐sims and the RO‐CT average values range between −1.1% and 1.2% for target coverage and within 0.8% for OAR and V80% metrics. Similarly, the sCT and ethos CBCT differences are within ±1.1% of the OAR and coverage metrics.

**TABLE 1:** Average RO‐sim dose metrics compared against dose metrics extracted from VMAT treatment plan recomputed on each of the listed CT or CBCT images.RO‐CT‐1 is Siemens X.cite and RO‐CT‐2‐4 is siemens definition AS.

	Reference RO‐sim average dose metrics						
	Target volume (cc)	Target D98% (cGy)	Target D95% (cGy)	Target D2cc (cGy)	Small bowel D2cc (cGy)	Large bowel D2cc (cGy)	V80% (cc)
	35	3136	3158	3541	588	661	67

	% Difference from reference value [%]						
	Target volume	Target D98%	Target D95%	Target D2cc	Small bowel D2cc	Large bowel D2cc	V80%
RO‐CT‐1	0.1	−1.1	−0.2	−0.2	0.2	−0.8	−0.2
RO‐CT‐2	−0.1	0.0	0.5	0.0	−0.2	−0.5	0.0
RO‐CT‐3	0.1	0.0	0.5	0.1	−0.3	0.5	0.1
RO‐CT‐4	−0.1	1.2	−0.9	0.1	0.2	0.8	0.1
PET/CT‐1 (120 kVp)	−0.1	−1.1	−0.4	0.1	0.2	−0.6	−0.3
PET/CT‐1 (140 kVp)	0.1	−0.7	0.2	0.4	0.2	−0.8	0.5
PET/CT‐2 (120 kVp)	0.1	−1.1	−0.3	−0.2	−0.2	−0.6	−0.3
PET/CT‐2 (140 kVp)	0.1	−1.1	−0.1	0.0	0.0	−0.6	−0.1
Ethos sCT	0.4	−1.1	−0.4	−0.4	−0.7	−1.0	−0.9
Ethos HS CBCT	−0.1	−1.0	−0.3	−0.5	−0.5	−0.8	−0.8

### 3.3 Clinical implementation experience

The DtT workflow was applied on an initial three patient cohort with metastatic osseus targets. An initial virtual consultation was done to explain and coordinate the DtT treatment. Based on target position, bespoke lateralized fields were configured for optimization and avoidance of potentially out of CBCT field‐of‐view regions. Static IMRT versus VMAT was evaluated when target complexity warranted the potential improvements with VMAT plans.

Table 2 provides the target, prescription, plan, and initial adaptive fraction time details for the three patients. The initial patient took the longest amount of time, with increased contributions during the initial setup and plan review stages, while the subsequent patients ranged between 37.2 and 40.9 min of total time‐on‐couch. VMAT was beneficial only for the sacrum target (patient 3) where the total number of MUs in the reference plan were reduced by over 50% and the computational time increased from 1 to 6 min compared with the static field IMRT plan.

**TABLE 2:** Patient and site‐specific clinical details and adaptive plan MUs and treatment times. Of the four sites, one was treated with a two arc lateralized VMAT plan. Times are extracted based on DICOM timestamps and automatically recorded time‐points in the ETM (no manual time tracking was performed).

Patient	Site	Low Rx (cGy)	High Rx (cGy)	Fx	Plan type (Laterality)	MU ref	MU adapt	Setup time (min)	Image review + contour time (min)	Plan calc time (min)	Plan review time (min)	Total time (min)
1	scapula	2400	3600	3	9 field IMRT (Left)	2654	2626	16.7	3.2	0.9	11.3	50.9
2	iliac	1600	2400	1	9 field IMRT (Right)	6275	6435	13.0	5.9	0.8	2.9	38.5
3	scapula	−	3900	3	9 field IMRT (Left)	3407	3750	15.0	4.8	1.0	4.3	37.2
	sacrum	2700	3600	3	2 arc VMAT (Right)	6213	6200	6.3	8.6	5.6	9.2	40.9

**FIGURE 4:**
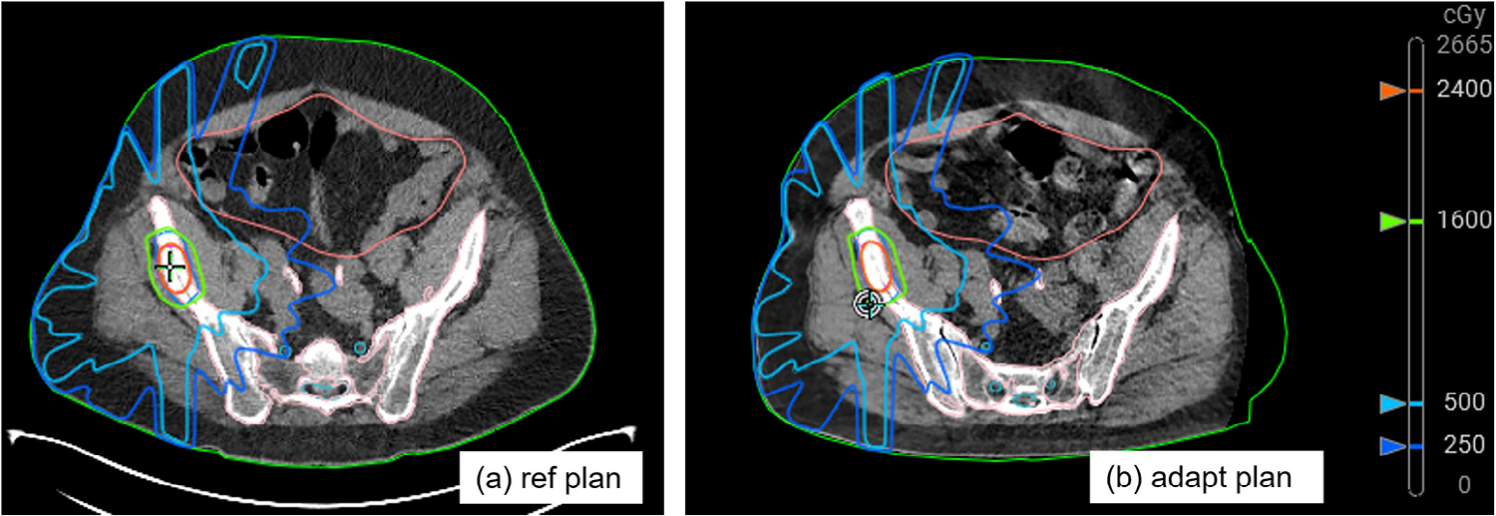
Patient reference plan on the reference diagnostic PET‐CT (a) and the adapted plan on the Ethos HS CBCT (b).

Figure 4 provides an image of the reference and adapted plans for Patient 2. The change in patient curvature is visible on the PET‐CT couch (Figure 4a). Further, a discontinuity is present in the patient external contour due to the limitation of the CBCT field‐of‐view (Figure 4b), and the selected beam arrangement avoids this region. Table 3 provides the comparison of these dose distributions via 3D gamma (2%/2 mm) and D95% for the PTVs.

**TABLE 3:** sCT and HS CBCT dose comparison via eclipse using D95% and gamma pass rate (2%/2mm) metrics.

			D95%			
Patient	Site	Structure	CBCT	sCT	%Diff (sCT – CBCT)	Gamma Pass Rate (%)
1	Scapula	PTV2400	2723.9	2752.7	1.06	95
		PTV3600	3581.6	3641.9	1.68	
2	Iliac	PTV1600	1917.2	1915.9	−0.07	98.7
		PTV2400	2430	2434.4	0.18	
3	Sacrum	PTV2700	2364.3	2354.1	−0.43	95.7
		PTV3600	3375.6	3351.1	−0.73	
	Scapula	PTV3900	3883	3917.8	0.90	99.4

Of the patients, one was treated for two sites, and another underwent single fraction treatment. Overall, three plans were transitioned from adaptive to IGRT. The time for preparing this transition ranged between 1.3 and 4.2 h. The total on‐unit times for the IGRT fractions were 27 and 21 min for patient 1, 7, and 11 min for the scapula site for patient 3, and 12 and 20 min for the sacrum target for patient 3.

## 4 DISCUSSION

The implemented DtT workflow demonstrated applicability of diagnostic image‐based planning for muti‐fraction SBRT treatments by using an online ART system. This work extends single adaptive fraction workflows applied within the palliative regime^13^, ^16^ to longer course SBRT treatments. Site specific end‐to‐end testing plays a key role in establishing a functional adaptive DtT clinical program. Careful patient selection, image review, and beam geometry specification are key in ensuring a successful process. By leveraging a single adaptive fraction to establish subsequent non‐adaptive sessions, the total on‐table time and staff resources could be reduced by using the comparatively faster standard IGRT workflow. It was found that the lack of integrated mechanisms for transitioning from adaptive to conventional IGRT workflows necessitates introduction of in‐house solutions for efficient contour and plan transfers.

Validation focused on ensuring HU and dose computation accuracy across imaging systems. Institutions must verify that incoming diagnostic images are sufficient for accurate dose computation since sCT relies on reference image HU values. The Ethos HS imaging system showed improved HU accuracy over standard CBCT, with iCBCT Acuros reconstructions aligning with the RO simulation HU range. These findings align with Robar et al.^23^ who reported median HU differences of ‐0.8 HU for breath‐hold and ‐10.5 HU for free‐breathing between CT simulators and the HS system. Dosimetric evaluation of PET‐CT, sCT, and HS CBCT images revealed coverage, near‐max, and volumetric metrics within ±1.1% of RO‐CT averages, consistent with the agreement found by MacDonald et al. among dose computed on Hypersight CBCT or fan‐beam CT and ion chamber measurements.^24^ HU variability, registration uncertainty, and contour propagation differences contribute to these dose metric variations. Additional dose uncertainties, such as a 1%–1.7% impact on the PTV D95% (Table 3) observed during the first patient adaptive treatment, highlight the importance of vigilant staff in identifying and evaluating dosimetrically significant deviations. Future ETM upgrades will allow direct on‐CBCT dose computation, removing the need for HU validation of reference CT and addressing patient external deviations within the CBCT field‐of‐view. Field‐of‐view limitation will still require the use of lateralized or bespoke field arrangements. Additionally, CBCT imaging artifacts, particularly in patients with high‐density implants or gas, will become critical considerations in both DtT and non‐DtT adaptive workflows.

For the initial three DtT patients, adaptive fractions lasted 50.9 min for the first patient and 38.5–40.9 min for subsequent ones, comparable to times reported in the FAST‐METS study^13^ and MacDonald et al.’s testing.^24^ These times include patient consent and setup. Contouring took less than 8.6 min, with most completed within 5 min, facilitated by treatment of osseous targets and proximal OARs. Post‐first fraction IGRT treatments ranged from 7 to 27 min, with significant time reductions after the first patient. Overall, efficiency improved as staff gained experience with the DtT workflow.

A limitation of the DtT process was the effort required when transitioning from adaptive plans to IGRT. Online ART can be more costly than conventional treatments, motivating a switch to non‐adaptive treatments following the first session. Running all fractions as online ART simplifies the multi‐fraction DtT process but increases individual fraction time. Given the short active adaptive time (10–20 min for contouring and plan review), an all‐adaptive DtT workflow is feasible. Within the current ETM version the transition to from adaptive to IGRT is time intensive for all team members. The greatest time expenditure was in exporting and preparing the adaptive CBCT, contours, and beam set for Eclipse import. The subsequent version of Ethos (2.0MR1) would eliminate this by providing integrated tools to use the adaptive fraction's HS iCBCT Acuros CBCT image and contours to generate a new plan for subsequent IGRT fractions. Transitioning to IGRT should be evaluated by the team based on need for daily OAR sparing and anticipated target variations, and MD availability to support adaptive treatments for subsequent fractions. While this study focused on bony targets, soft tissue targets without motion management needs could also be treated with DtT, and those with expected intra‐fractional variations would benefit from adaptive DtT fractions. Future tools allowing conversion from adaptive to IGRT fractions will greatly enhance DtT workflow efficiency, enabling patient‐specific decisions between IGRT and adaptive fractions. Current DtT workflows do not include target motion assessment or respiratory motion management, as these are not performed during diagnostic imaging. Additionally, this study involves only three patients; larger cohort studies would better assess workflow performance and patient impact.

## 5 CONCLUSION

A DtT workflow using a single adaptive fraction for multi‐fraction SBRT has been successfully implemented using ETM. The conversion to non‐adaptive treatments following the first fraction requires vendor integrated tools to improve efficiency. This process reduces the need for on‐site patient presence prior to radiation treatment and has the opportunity to reduce diagnosis to treatment times and sets the groundwork for future applications beyond boney metastatic targets, especially for patients from rural parts of the country who live further away from radiation oncology centers and those that have increased challenges with transportation to attend multiple appointments.

## AUTHOR CONTRIBUTIONS

All co‐authors contributed to manuscripts review and data collection. V.N.M., S.S.P., and J.A.K. additionally contributed through investigation design, data analysis, and manuscript generation.

## CONFLICT OF INTEREST STATEMENT

None of the authors have a conflict of interest to declare except for J.A.K. who received travel and speaker honorarium from Varian (Siemens Healthineers) for work unrelated to this manuscript.
